# Autistic Women’s Experience of Motherhood: A Qualitative Analysis of Reddit

**DOI:** 10.1007/s10803-024-06312-7

**Published:** 2024-04-26

**Authors:** Sandra Thom-Jones, Imogen Melgaard, Chloe S. Gordon

**Affiliations:** 1 Australian Catholic University Limited, Melbourne, VIC 3777 Australia; 2 School of Behavioural & Health Sciences, Australian Catholic University, Fitzroy, VIC Australia; 3 Institute for Positive Psychology and Education, Australian Catholic University, Fitzroy, VIC Australia

**Keywords:** Autism, Autistic women, Motherhood, Peer support, Reddit

## Abstract

Autistic mothers remain under-represented in parental and autism research despite the associated physical and psychosocial challenges that accompany the transition to motherhood. Extant literature suggests autistic mothers experience sensory difficulties, communication challenges, stigma, and comorbidities as difficulties, but these studies have focused on autistic women in the perinatal period. The aim of this study was to examine reflections on motherhood from a Reddit community for autistic parents. Identified themes were *Autistic Mothering is Different*, *Autistic Mothers Need Autistic Mothers, Autistic Mothers Experience Stigma*, and *Learnings from Lockdown.* Findings extend existing research by offering insight into the ways autism impacts mothers beyond the perinatal period and have important implications for the future design and delivery of support services for autistic mothers.

## Autistic Women’s Experience of Motherhood

There is limited empirical research on the experiences and needs of autistic women, particularly in salient lifespan periods such as motherhood. The American Psychiatric Association (American Psychiatric Association, 2022) defines autism spectrum disorder as a neurodevelopmental disorder, characterised by impairments in social interaction and communication, accompanied with restricted and repetitive behaviours. Despite heterogeneous presentation (Hollander et al., 1998), autism has been profiled as a condition that largely affects males (Loomes et al., 2017). The use of predominantly male samples in studies (Webster & Garvis, 2017), bias around gendered readings of symptomology (Ronald et al., 2006) and theories such as extreme male brain (Baron-Cohen, 2002) have contributed to this profile with diagnosis rates four times more likely for males than females (Halladay et al., 2015; Rivet et al., 2011). There is a sizable body of research that focuses on diagnosis, supports and interventions for autistic children, with considerably less focus on the outcomes of autistic adults (e.g., Eldevik et al., 2009; Johnson & Myers, 2007; Landa, 2018; Trevarthen, 1998). Females who are diagnosed tend to have more severe symptoms or receive their diagnosis later than equivalent males (Giarelli et al., 2010). As a result, little is known about the experiences and needs of autistic women.

Researchers have begun to investigate autistic women’s experience in the perinatal period (e.g., Donovan, 2020; Gardner et al., 2016; Hosozawa et al., 2021; Pohl et al., 2020; Rogers et al., 2017; Sundelin et al., 2018), but there is still much unknown about autistic women’s transition into early motherhood and beyond. Factors impacting motherhood have broad-reaching and dyadic ramifications for psycho-social and physical health outcomes and warrant further investigation (McDonnell & DeLucia, 2021). Existing studies of autistic women in the perinatal period highlight a range of unique experiences. These include sensory sensitivity (Gardner et al., 2016; Rogers et al., 2017), difficulties with change and challenges with communicating (Donovan, 2020; Gardner et al., 2016), and increased rates of perinatal depression and anxiety (Pohl et al., 2020). Apart from the study by Pohl et al. (2020), this body of research does not address whether these experiences continue into later stages of motherhood, nor does it offer insight into the strategies employed by autistic mothers to manage these challenges. The experience of features typical to autism have not yet been studied in autistic mothers in later stages of motherhood including mothers of toddlers, children, adolescents, and (young) adults.

The discomforts of pregnancy combined with the hyper/hypo sensitive sensory responses typical in autism are suggested to result in a heightened discomfort for autistic women (Sundelin et al., 2018). Gardner et al. (2016) reported that participants characterised the impact of pregnancy and birth as a time of “enhanced sensitivities to touch, light, sound and interactions” (p. 32). These enhanced sensitivities are suggested to lead to negative experiences at medical check-ups, due in part to women’s heightened sensory processing, and perceptions of being judged as an autistic mother (Gardner et al., 2016; Rogers et al., 2017). Researchers did not address strategies to manage heightened sensory processing. The lack of empirical research investigating the impact of sensory responses in later stages of motherhood offers scope for further exploration.

There is limited research investigating the impact of other features of autism on autistic mothers, including social and communication difficulties and restricted and repetitive routines (American Psychiatric Association, 2022). A comparative study between autistic and non-autistic mothers by Pohl et al. (2020) addresses some of these concerns in a preliminary investigation. Participants completed an online survey responding to questions reflecting on their experience of motherhood. Findings indicated autistic mothers reported greater difficulties with the demands of parenting, such as the need to multitask and provide socialising opportunities for their child, as well as significantly more difficulty and hesitation in communicating with services. Participants in this study were largely mothers to adolescents and the data collected were reflections on earlier stages of motherhood. Pohl et al. (2020) did not include the qualitative data from open-ended questions in the survey. As such, there was no reporting on the qualitative experiences of autistic mothers.

Communication difficulties experienced by autistic people are defined as deficits in social-emotional reciprocity, nonverbal communicative behaviours, and management of relationships (American Psychiatric Association, 2022). In a qualitative study of 24 autistic birthing women, Donovan (2020) reported communication challenges as a key theme that affected participants in both daily life and in their experience of health care. Participants’ experience of labour was negatively impacted by the stress of a new or overly stimulating environment, their idiosyncratic verbal communication styles, difficulty reading social cues, and a tendency to interpret communication literally. Similar sentiments were expressed by participants in the study by Gardner et al. (2016) who reported feeling unheard and judged by others as they navigated early parenting. These findings are similar to those of Aylott (2010) and Nicolaidis et al. (2015) who identified stigma associated with autism and the lack of specialised knowledge and training amongst service providers as additional important factors impacting communication challenges for autistic adults. Understanding how stigma and communication challenges are experienced by autistic mothers is needed.

Existing studies recommend health professionals develop a deeper understanding of autism to better meet the needs of autistic mothers (Donovan, 2020; Gardner et al., 2016; Pohl et al., 2020). This has heightened imperative due to the caregiving responsibilities of mothers. Little is known about the ongoing impacts of sensory sensitivity for autistic women in later stages of parenthood, how autistic mothers experience communication difficulties in other settings such as their child’s social community and what impact stigma has on their parenting role. Furthermore, there is limited research that investigates the unique strengths and cognitive skills that often accompany autism (Meilleur et al., 2015).

Information about the experiences of being an autistic mother can be found online (Bargiela et al., 2016; Litchman et al., 2019). There is a growing body of autistic mothers raising issues most pertinent to them (e.g., PurpleElla, 2017; YoSamdySam, 2019). Until now, this rich source of information shared online has been largely uncaptured in academic literature. These voices add to empirical studies that highlight risk and negative outcomes for autistic mothers (e.g., Hosozawa et al., 2021; Sundelin et al., 2018) by offering first-person testimonial and peer discussion about shared challenges.

Peer support is a strategy used to assist individuals facing circumstances that differ from a dominant norm by offering them support from others with similar lived experience (Mead et al., 2001; Shalaby & Agyapong, 2020). Studies investigating the use of peer support with autistic individuals have largely focused on children (e.g., Carter et al., 2017; McCurdy & Cole, 2014), however the findings of Rosqvist (2019) suggest that there is utility in informal autistic peer supports to promote abilities and interests amongst adults. Reddit is a social media platform that offers online peer support where users may anonymously discuss specific topics of interest in subject specific subreddits. Recent reviews have found large numbers of studies on the use of Reddit to explore sensitive topics such as illicit substance use (Chi & Chen, 2023) and anxiety and depression (Boettcher, 2021). Results from these and individual studies have provided support for the view that analysis of Reddit posts can facilitate the collection of data on behaviour patterns and experiences of hard to reach groups (Chi & Chen, 2023), as well as facilitate the development of appropriate messaging and dissemination strategies for public health interventions such as vaccine uptake (Melton et al., 2021).

In a study that examined a decade of content on Reddit parenting boards, Ammari et al. (2019) found that parents are more likely to discuss stigmatising topics anonymously. In addition, these anonymous testimonials offer users access to shared experience and support. Qualitative studies of Reddit content have been used to investigate the experiences of fathers (e.g., Pilkington & Rominov, 2017; Teague & Shatte, 2021), but no studies appear to have used Reddit data to investigate the experiences of autistic mothers.

The aim of this study was to capture autistic mothers’ reflections on the experience of motherhood and to investigate the support they offer each other through an inductive qualitative analysis of the content in an autistic parenting subreddit. Investigating the needs and concerns of autistic mothers is in keeping with the Australian Government’s National Standards for Mental Health Services (2010) that encourages consumer voices be involved in planning delivery and evaluation of services. The purpose of this project is to contribute knowledge that increases awareness and understanding of autistic mothers, so that they may receive future targeted research and support.

## Methods

### Study Design

A qualitative exploratory approach was applied to secondary data scraped^1^ from a subreddit, *r/AutisticParents* on the social media site Reddit. Before commencing the study, a PsycInfo search of studies utilising data from Reddit was conducted. It retrieved 161 peer reviewed studies in other research areas (e.g., Mason et al., 2021; Sowles et al., 2018;), supporting Jamnik and Lane’s (2017) endorsement of the quality of data that can be obtained from online sources. Reddit was selected as an appropriate social media platform due to the site being a public discussion platform in which posts and comments are not limited by word count. In addition, there is a requirement for registration, but users can remain anonymous. All content being shared publicly is with user knowledge. These features differ from platforms such as Twitter and Facebook, where the format, settings and end user agreement may preclude in-depth and ethical study of content. Data collected for this study was pre-existing, non-identifiable and publicly available. As such, this study was classified by the [blinded for review] University’s Human Research Ethics Committee as a low-risk study.

Content discussing autistic experiences of mothering was analysed to gain insight into the issues and experiences of autistic mothers. Community guidelines outline that the subreddit is for autistic parents to discuss the unique challenges and perspectives of being an autistic parent. Guidelines also invite non-autistic parents of autistic children to participate so they may better understand their child.

### Community Involvement and Researcher Positionality

Consistent with the research team’s commitment to participatory research, and the importance of own voices driving the research agenda, a series of steps were taken to ensure autistic involvement in the study. The research team included an autistic autism researcher, and autistic people were consulted at all stages of the research. Two members of the university’s Autism Peer Review Panel (autistic academics not involved in the study) reviewed the manuscript prior to submission. As per Savin-Baden and Major’s (2013) recommendations for positionality, it is important to locate the position of the author in relation to the subject, participants, and research context. The first author is an autistic autism researcher who is also a parent of autistic people. The second author is a female student at a Western university who is mother to an autistic child and works in advocacy and mental health support for new parents, and the third author is a non-autistic qualitative researcher and new mother who has co-authored studies on the experiences of autistic people.

### Data Collection

All posts from *r/AutisticParents* were retrieved from the community’s inception in May 2018, with last date of retrieval on 30 July 2021. Retrieval of data was performed using the Reddit Application Programming Interface and archived in JavaScript Object Notion format. Attributes of retrieved data for posts included: username, title, body, date created, and URL. Attributes for comment data included: username, subreddit ID, body, date created and URL.

The inclusion criteria were: (1) written reference to the writer’s own experiences of motherhood; and (2) statement or inference of the writer’s autism. Representative excerpts of inclusion criteria are presented in Table 1. Quotes selected for use were carefully chosen so that they communicated necessary themes but did not contain identifying information.

**Table 1:** Representative Excerpts of Inclusion Criteria

Inclusion Criteria	Representative Excerpts
1. Written reference to experiences/questions about motherhood	“I realised it wasn't just "maternal hormones"”
	“I'm a single mom and in addition to autism”
	“I'm a 26 yo female with a toddler”
	“I feel like a terrible mother”
	“I had hyperemesis gravidarum”
	“I have been trying to connect with other mums”
2. Statement or inference of the writer’s autism	“I didn’t know yet that I was on the spectrum”
	“In addition to autism I have…”
	“I'm an autistic mum”
	“I have recently found out I'm autistic.”
	“At the time I didn't know I was autistic”
	“…how exhausting trying to mask as a neurotypical person”

### Data Cleaning and Preparation

The archived data were converted from JavaScript Object Notion format and reconstructed into plaintext files, where each text file represented a Reddit post and all associated comments.

This was performed to conduct selection, coding, and analysis in the NVivo software package (released in March 2020). Given the relatively small number of posts collected (*N* = 89), no automated filtering was performed to remove non-relevant items. The collected posts were systematically sorted into the following cases: included posts, included comments, and excluded non-relevant data. A total of 66 posts were excluded in the initial stages of data familiarisation for not meeting the inclusion criteria. These comprised posts and comments that were written by non-autistic parents of autistic children, contained no clear reference to being a mother, or were invitations to participate in research.

### Data Analysis

Data were imported into NVivo software (released in March 2020) for management. The selected posts and comments were read and re-read for familiarisation with the data and to identify the content that related to autistic motherhood.

An inductive approach to data analysis was utilised, following Braun and Clarke’s approach to reflexive thematic analysis (Braun & Clarke, 2014, 2019). This approach was chosen because the aim of the study was to understand the experiences of autistic mothers from their perspectives. This paradigm recognises that the researchers’ own lived experiences will influence interpretation of the data.

The initial coding was undertaken by the second author, and reviewed by first and authors for resonance from their perspectives as an autistic parent and a non-autistic new mother respectively. The initial 33 codes and their content were discussed among the research team to review and refine definitions and application to the data. These were further refined and combined to eliminate overlapping codes and ensure that all included text was relevant, resulting in a total of 18 codes. This was primarily the merging of overlapping codes (such as, combining ‘sound sensitivity’ and ‘light sensitivity’ under the code ‘sensory difficulties’, ‘camouflaging and ‘masking’ under the code ‘masking’) and excluding codes unrelated to parenting.

After manual coding, examination of the NVivo Hierarchy Chart was used to identify significant patterns present in the data. This step facilitated discussion to identify the initial themes. Codes that included reference to characteristics that were consistent with diagnostic criteria for autism (American Psychiatric Association, 2022) and the impact this had on mothering were assigned to the theme *Autistic Features*. A further four themes were developed by IM: *Autistic Mothering is Different, Autistic Mothers Need Autistic Mothers, Autistic Mothers Experience Stigma*, and *Learnings from Lockdown.* These themes were reviewed by the research team and checked against the dataset to ensure they represented the central experiences present in the data. The five initial themes were also refined so that *Autistic Features* was integrated as a subtheme in *Autistic Mothering is Different*. Once the themes were reviewed and revised, the underpinning meaning was explored to ensure each theme was appropriately named and defined to accurately capture and analyse the narrative derived from the data.

## Results

A total of 23 posts and 131 related comments were coded and analysed. Of these, 53 data files explicitly included the terms: mom, mum, mother. The 23 posts (5963 words) were made by 23 unique users and averaged a mean length of 259 words. These posts were independently screened by the first and second authors to confirm that they were posts by autistic mothers. This included ensuring there was a direct or inferred reference to being a mother (e.g., “I’m a single mom” (post 1), “I am a mother of 2” (post 10), “I feel like a horrible mother” (post 23)) and to being autistic (e.g., “Should I tell anyone I’m autistic” (post 2), “..dealing with NT moms…I mask like I breathe” (post 9), “I’m 25 and I’m autistic” (post 17″)). Twenty of the 23 posts included information on the gender of the poster’s child/ren, and 18 included information on the age of their child/ren, although this should be interpreted with caution as some may have had other children they did not mention in the post; the majority (15 of the 18) were referring to children aged 5 and under (see Table 2).

**Table 2:** Details of Children Mentioned in Posts

Post #	# children	Gender	Age
1	1	Male	4 years
2	1	Male	5 years
3	1	Female	2 years
4	1	Female	3 years
5	1	Female	3 years
6	1	Male	Toddler
7	1	Male	4 years
8	1	Male	Toddler
9	1	Female	3 years
10	2	N/S	School-aged
11	1	Female	School-aged
12	2	1 Male	3 years
		1 Female	1 year
13	N/S	N/S	N/S
14	2	Male	N/S
15	> 1	Oldest is female	N/S
16	1	Male	3 years
17	1	Male	2 years
18	1	Female	N/S
19	1	Male	15 years
20	N/S	N/S	N/S
21	1	Male	5 years
22	1	Male	5 weeks
23	2	1 Male	5 years
		1 Female	3 years

The 131 comments (16,999 words) averaged a mean length of 130 words. These were made by 65 unique users with an additional six comments made by deleted users. It is unknown if these comments originate from a single user or multiple users. There were 27 data files that included reflections on the experience of pregnancy and although these supported the findings in research literature, they were excluded from analysis for not meeting the aims of this study. These 131 included comments were independently screened by the first and second authors to confirm their relevance.

### Themes

The four themes – *Autistic Mothering is Different*, *Autistic Mothers Need Autistic Mothers, Autistic Mothers Experience Stigma*, and *Learnings from Lockdown* – their definitions and representative examples are presented in Table 3.

**Table 3:** Themes, Definitions, Frequency and Excerpts

Theme	Definition	Representative Excerpt
*Autistic Mothering is Different*	Perspectives, strengths, and difficulties experienced as an autistic mother	“Stimming will always be encouraged in this house, as are special interests, repetition, and anything else that makes my son or me feel better.”
		“I still struggle to forgive myself that I haven’t been able to act like a NT mom.”
		“It also can sometimes lead to meltdown which I usually try to hide in the other room if it happens.”
		“Child development is one of my special interests.”
*Autistic Mothers Need Autistic Mothers*	Self-disclosure, validation, normalisation, and empathy towards the experiences of fellow autistic mothers	“Just remember you are not a bad person, lots of people struggle with the same thing.”
		“I can relate, you sound a lot like me!”
		“Yep, I’ve been there. My son is 2.5 now”
		“Oh wow you put into words how I feel exactly.”
*Autistic Mothers Experience Stigma*	Experiences or anticipation of receiving stigma for being an autistic mother	“Autism has allowed me to love myself a little better.”
		“if I seem weird to other parents, they'll be less likely to invite us to their kids’ parties”
		“For the past 2 years we’ve stopped pretending we’re a “normal” family and everyone is much happier for it.”
*Learnings from Lockdown*	Ways in which the pandemic has impacted the experiences of autistic mothers	“Can we all maintain 6 feet of distance forever?
		“thanks to covid I’ve been working from home the whole pregnancy, and can just zone out or nap off the overstimulation if I need to”

### Autistic Mothering Is Different

This was the predominant theme, with a total of 234 references across the dataset. These posts and comments expressed unique understandings of being an autistic mother (e.g., “I feel like I can read and communicate with my kids more seeing how verbal communication isn’t my primary method of communication” and “I could use my weaknesses to explain and teach them about myself and the world around me”). The theme comprised three subthemes: *Autistic Features*, *Difficulties* and *Strategies*.

*Autistic Features* incorporated all mentions of experiences that aligned with an autism diagnosis (American Psychiatric Association, 2022) such as “Turns out, most of the time I'm just in sensory overload and need time out to calm down” and “the most difficult thing is if one of the kids starts crying or shouting because the room echoes.” Experiences relating to sound and touch were frequently discussed.

*Difficulties* related to the specific challenges of raising a child and managing autism and often included statements of regret or guilt (e.g., “I am very aware of my shortcomings regarding my ability to guide my sons in the outside world” and “I am also almost constantly overwhelmed and worry about them bearing the brunt of my anxiety and lack of family support”). Contributors wrote with self-awareness of their limitations in social communication as well as their struggle to stay regulated in front of their children.

*Strategies* recognised solution focused experience sharing such as “When I’m on the verge of a sensory ‘meltdown’, I escape for a few minutes to ground myself” and “I instituted a very early bedtime… so that I could have completely touch-free time to myself each night.” Solutions were oriented to managing sensory stimulation and navigating social engagements.

#### Autistic Mothers Need Autistic Mothers

This was the second most prominent theme, identified 145 times across the datset. It comprised comments that validated and normalised autistic mothers’ experience and included instances of empathy, advice giving, experience sharing, advice seeking and educating. Posts included more instances of the advice seeking and were catalysts for discussion and experience sharing e.g.,

“Advice needed: We're going to a birthday party”

“If you're autistic and have been pregnant, what was it like for you?”

“Has anyone been able to modify a stim themselves or know of any techniques?”

“How do you deal with the school run?

Comments included more instances of advice giving and expressions of empathy and were frequently solution focused e.g.,

“One thing that helped me when my daughter was screaming a lot is vibes ear plugs.”

“For me it's remembering to manage my other conditions, taking short breaks, and having things like a weighted blanket and stim toys to manage sensory stuff.”

Peer support was observed between posts that sought advice and comments that responded with validation, shared experience, and support e.g.,

Post: “Do other autistic parents feel this way by the end of the day? I’m a 26 yo female with a toddler… I feel so "not me" by the end of the day.”

Comment 1: “Same boat! My son is six months and I love him So Much but he also triggers my overload-sensory stuff a Lot.”

Comment 2: Don't beat yourself up about feeling exhausted, some parents feel that way even though they're NT and have no particular mental health issues. You're doing fantastic, all things considered.

Comment 3: “I hear you □”

Interesting, but less frequent, were comments that sought to educate. These were incorporated into this theme for their role in advocating and destigmatising autism and occurred in response to posts made by parents of autistic children who had mistakenly posted material deemed offensive by the autistic community. Examples of educating included “First off please try to stop thinking (of) it as high functioning and using words like normal” and “We stim to help release energy, be it good or bad energy.”

#### Autistic Mothers Experience Stigma

Several contributors described some degree of stigma they had encountered or feared encountering. This theme, with a total of 28 references coded, included the subtheme *Revealing Diagnosis*. This theme revealed some of the pressures specific to being an autistic mother as well as moments of contributors embracing their autistic identity e.g.,

“There's added pressure, because if I seem weird to other parents, they'll be less likely to invite us to their kids' parties or let their kids come to our house.”

“Most adults do not understand autism (unless they know someone with it) and will make negative assumptions because they don't understand.”

“Being an autistic mother is isolating, not that autism itself isn't isolating, but even more so.”

“For the past 2 years we've stopped pretending we're a "normal" family and everyone is much happier for it”

“Autistics can be great parents too, although we might face different challenges than NTs - it is still rewarding!”

#### Learnings from Lockdown

This theme was unanticipated in the research aims of this study, but unsurprising given the time in which data were collected. Whilst only a minor theme, with 10 references coded, it contained interesting perspectives from autistic mothers about how elements of lockdown made life *more* manageable (e.g., “I love that right now, it is totally normal and expected to decline social invites” and “some things about COVID lockdown I really enjoy”). This aligned with literature that outline sensory overload and social and communication difficulties for autistic people and reflected how challenging the management of this in daily life can be.

## Discussion

The aim of this study was to investigate online discussion between autistic mothers to explore how they wrote about their experience of autism and motherhood. This was achieved by inductively analysing the content autistic mothers posted in a Reddit community for autistic parents. Four themes emerged: *Autistic Mothering is Different*, *Autistic Mothers Need Autistic Mothers, Autistic Mothers Experience Stigma*, and *Learnings from Lockdown*. The data helped to explore the ways contributors’ autism influenced their experience of mothering. In addition, it provided insight into the ways autistic mothers support each other online. Autistic mothers discussed diverse experiences and a range of challenges. Contributors shared solutions and validation through many stages of motherhood and reflected a heterogeneous presentation of autism (Hollander et al., 1998) and of motherhood. However, commonality was found in discussing the challenges that occur at the intersection of contributors’ autism and role as mother. Overall, findings indicated that *r/AutisticParents* provides opportunities for autistic mothers to seek informal peer support and share experiences. This study adds to the small body of research that examines autistic women in motherhood offering insight into how online communities support and validate their experience. Findings have important implications for future design and delivery of support options for autistic mothers.

Previous research indicated autistic mothers in the perinatal period experience social and communication difficulties, and stigma (Donovan, 2020; Gardner et al., 2016; Pohl et al., 2020; Rogers et al., 2017; Sundelin et al., 2018). Experiences of social and communication difficulties were observed in the data, but these difficulties were solely discussed in relation to managing interactions with other parents, which differed from the findings of Donovan (2020), Gardner et al. (2016) and Pohl et al. (2020) who focused on communication difficulties with health professionals. This is unsurprising given this study did not focus on birthing women who are regularly interacting with staff in health care settings, but important nonetheless as analysis of discussion between autistic mothers offers a unique way to capture their day-to-day concerns. Contributors seemed aware of their social and communication challenges and spoke of how tiring the act of masking is as a strategy to compensate for this. Experiences of masking, whereby individuals both consciously and unconsciously suppress their natural autistic responses (Pearson & Rose, 2021), is recognised in emerging literature as a costly and frequently harmful strategy autistic individuals adopt to fit in and avoid stigma (Raymaker et al., 2020). This was an important finding from this novel data as these difficulties are not discussed in depth in the existing literature on autistic mothers but may come at great cost.

While discussions of stigma were observed in the data, they were not related to interactions with health professionals as had previously been reported by Aylott (2010), Gardner et al. (2016), Nicolaidis et al. (2015) and Pohl et al. (2020). Discussions about stigma largely related to contributors seeking advice about sharing their diagnosis with other people within their child’s social community. These occurred in only a small portion of the data and related more specifically to discussions of autistic identity and self perception rather than direct experiences of discrimination. Contributors seeking advice were looking for solutions to explain to others what they perceived as their *‘*weird’ behaviour and expressed a desire not to stand out from other parents. Further exploration of the ways autistic mothers perceive and experience stigma in their daily lives would be useful to help determine if the dominant medical model of disability and ‘otherness’ marginalises their experience and overlooks difference and unique strengths.

The discomforts of sensory stimulation and experiences of sensory overload or meltdown were frequently discussed in the data. Reflections on the experiences of pregnancy implied that heightened sensory sensitivity in pregnancy appears additionally challenging to autistic women. These insights were not included due to the scope of this study but supported the findings of Donovan (2020) and Gardner et al. (2016). Other posts about sensory sensitivity related to discussions about the demands of mothering, particularly the noise and busy nature of contributors’ children. While posts identified the challenges of sensory overload, comments were oriented toward sharing solutions such as noise reducing headphones and sensory calming strategies. Contributors’ discussion around meltdown reflected an awareness of the impact this may have on their children and comments offered experience sharing and management strategies to prevent ‘meltdown’ in front of children. This pattern in the data supported Pohl et al. (2020) who found that there was no difference in autistic and non-autistic mothers’ ability to put the needs of their child above their own. However, it raises concerns about the long-term impact on autistic mothers of putting their children’s needs before their own when in a state of meltdown; for example, the increased likelihood of progressing to autistic burnout due to the ongoing emotional and sensory overload. In trying to avoid autistic burnout, Raymaker et al. (2020), found that autistic adults need acceptance, social support, and reduced expectations. How this is recognised and then achieved by and for autistic mothers is not yet addressed in research literature.

Hosozawa et al. (2020) found a lack of social support partially explained self-reported experiences of child mistreatment by autistic mothers of new babies. The findings of this study gave no indication of child mistreatment, but it is interesting nonetheless to recognise the importance of social support as a protective factor. Social support was evident in moments when contributors offered opportunities for direct messaging or shared sentiments for more active use of the subreddit. Pohl et al. (2020) found that autistic mothers experience motherhood as more isolating than their non-autistic peers. Explicit discussion of feeling isolated occurred only once in the data but was also implied in discussions of stigmatising topics especially when contributors included statements such as “Anyone else been there?” Contributors reaching out online suggested they could not draw on support or knowledge from their offline contacts. While social support is important for all parents, there is perhaps particular need for those who are vulnerable.

Informal peer support occurred frequently in the data and included contributions that shared, normalised and, validated strengths, challenges, and experiences within this subreddit (Mead et al., 2001). Advice seeking was commonly found in contributor posts and was a catalyst for experience and strategy sharing in comments. This supports a growing body of literature about online parent peer support (Ammari et al., 2019; Pilkington & Rominov, 2017; Teague & Shatte, 2021). Furthermore, it suggests that there may be utility in specialised peer support groups developed by and for autistic mothers. However, while peer support was evident in the data there are several limitations on the conclusions that can be drawn from this. This study was not able to assess contributors’ appraisal of the effectiveness of this support nor was it able to assess the outcomes for contributors and their children. Additionally, the response time between posts and comments was not factored into this research so it was unknown if responses to posts were immediate or delayed. Such information would enhance understanding and inform the design of future online peer support models.

The findings from this data offer insight that is informative for practice. Recognised peer support models of assistance in the perinatal period, including support for breast feeding (Forster et al., 2019) and postnatal depression (Biggs et al., 2015), could be expanded to include groups that cater specifically to the needs of autistic mothers. With further research and consumer consultation, these groups could extend beyond the perinatal period and come in the form of supported play groups or school parent groups that were able to support autistic mothers with managing sensory sensitivities and social and communication difficulties. Existing evidence on peer support demonstrates that it can successfully bridge the gap between consumers and health care professionals (Shalaby & Agyapong, 2020). Additionally, the benfits of online parent support groups (Ammari et al., 2019) and the findings of this study suggests an online model may have more positive utility with autistic mothers.

Study models that source data from Reddit are increasing the ways researchers can investigate consumer needs (Pilkington & Rominov, 2017; Teague & Shatte, 2021). Understanding the ways autistic features present and impact autistic mothers is important for health professionals to firstly understand and identify and secondly to help seek sustainable management solutions. Experiences such as masking, meltdown, sensory difficulties, and social and communication difficulties all offer insight into the ways caring for children can exacerbate autistic features. Although, mention of special interests and positive identity perspectives offer insight into the relative strengths of autistic mothers as they focus on child development and see parenting from a neurodiverse perspective that may intuitively meet the needs of their children in ways not afforded by a neurotypical perspective.

## Limitations

The nature of the data set limits the generalisability of the findings. All contributors had access to a computer, some level of (computer) literacy and wrote in English, which is not be representative of all autistic mothers in the population. Participants were included if they self-identified as autistic, which includes those without a formal diagnosis but also excludes those who have not identified as (or rejected a diagnosis of) being autistic. While the assessment of the 23 original posts against the inclusion criteria was objective (using specific terms to define inclusion), we acknowledge that the assessment of the 131 comments was more subjective. Many of the 65 users who made these comments explicitly stated their identity as an autistic mother, but others were implied by responding to a question posed to autistic mothers or in nuances in the wording of the comment that implied solidarity. We attempted to minimise the risk by the use of two independent coders, one a non-autistic psychologist and one an autistic parent and experienced autism researcher.

While advancing the research pertaining to autistic women, this study did not seek to address experiences of gender diversity. By focusing on women’s experiences of motherhood, this study assumed traditional family structures of care and therefore did not account for the experiences of step or queer parents. However, research indicates that autism is correlated with increased rates of unique expressions of gender identity (Dewinter et al., 2017; George & Stokes, 2018). How research incorporates the experiences of gender diverse and non-birthing subjects was beyond the scope of this study. Finally, the study did not distinguish between parents of children in different age groups, with the variation in issues and concerns that accompany different stages of child development.

## Future Research

Adjusting to parenthood is recognised as difficult and at times stressful. Many of the themes presented in the data were not dissimilar to non-autistic experiences of motherhood. However, many of the concerns raised, and much of the terminology used, resonated with known aspects of the autistic experience. Further study is needed to measure if and how experiences such as sensory overwhelm, fatigue, and stress in response to caring for children are quantitatively and qualitatively different from non-autistic motherhood. Conducting in-depth qualitative interviews that pose specific questions grounded in the insights described by this research would offer qualitative depth about the experiences of autistic mothers.

## Conclusion

The aim of this study was to capture the themes expressed by autistic mothers in social media comments about parenthood and to investigate the ways in which they supported each other. An inductive qualitative analysis of the content in the subreddit *r/AutisticParents* was conducted. The data revealed four key themes. The most prominent of these were discussions about the ways autistic features impacted contributors’ mothering, and the peer support they were able to offer each other. Online communities offer a rich source of qualitative data allowing researchers to analyse contributions made by consumer stakeholders. Increasing understanding equips professionals with a better chance of providing support that works with and meets the needs of autistic mothers in settings other than perinatal care. On the basis of this original data, it is recommended that further investigation be conducted into the applicability of peer support groups for autistic mothers. Additionally, further study into the impact of sensory and social communication challenges on autistic mothers is also recommended.

## Footnotes

1 ‘Data scraping’ refers to the process of using a computer program to extract data from another program (Marres & Weltevrede, 2013).
