# Correction: Autistic Women’s Experience of Motherhood: A Qualitative Analysis of Reddit

**DOI:** 10.1007/s10803-024-06457-5

**Published:** 2024-07-24

**Authors:** Sandra Thom-Jones, Imogen Melgaard, Chloe S. Gordon

**Affiliations:** 1 Australian Catholic University Limited, Melbourne, VIC 3777 Australia; 2 School of Behavioural & Health Sciences, Australian Catholic University, Fitzroy, VIC Australia; 3 Institute for Positive Psychology and Education, Australian Catholic University, Fitzroy, VIC Australia

**Correction to: Journal of Autism and Developmental Disorders**

10.1007/s10803-024-06312-7

The article “Autistic Women’s Experience of Motherhood: A Qualitative Analysis of Reddit”, written by Sandra Thom Jones, Imogen Melgaard and Chloe S. Gordon was originally published electronically on the publisher’s internet portal on 26th April 2024 without open access. With the author(s)’ decision to opt for Open Choice the copyright of the article changed on 10 July 2024 to © The Author(s) 2024 and the article is forthwith distributed under a Creative Commons Attribution 4.0 International License, which permits use, sharing, adaptation, distribution and reproduction in any medium or format, as long as you give appropriate credit to the original author(s) and the source, provide a link to the Creative Commons licence, and indicate if changes were made. The images or other third party material in this article are included in the article’s Creative Commons licence, unless indicated otherwise in a credit line to the material. If material is not included in the article’s Creative Commons licence and your intended use is not permitted by statutory regulation or exceeds the permitted use, you will need to obtain permission directly from the copyright holder. To view a copy of this licence, visit http://creativecommons.org/licenses/by/4.0. Open Access funding enabled and organized by CAUL and its Member Institutions.

